# Polysaccharide intercellular adhesin in biofilm: structural and regulatory aspects

**DOI:** 10.3389/fcimb.2015.00007

**Published:** 2015-02-10

**Authors:** Carla Renata Arciola, Davide Campoccia, Stefano Ravaioli, Lucio Montanaro

**Affiliations:** ^1^Research Unit on Implant Infections, Rizzoli Orthopaedic InstituteBologna, Italy; ^2^Department of Experimental, Diagnostic and Specialty Medicine (DIMES), University of BolognaBologna, Italy

**Keywords:** *Staphylococcus*, biofilm, ica locus, Polysaccharide intercellular adhesin (PIA), poly-β(1-6)-*N*-acetylglucosamine (PNAG), anti-PIA vaccine

## Abstract

*Staphylococcus aureus* and *Staphylococcus epidermidis* are the leading etiologic agents of implant-related infections. Biofilm formation is the main pathogenetic mechanism leading to the chronicity and irreducibility of infections. The extracellular polymeric substances of staphylococcal biofilms are the polysaccharide intercellular adhesin (PIA), extracellular-DNA, proteins, and amyloid fibrils. PIA is a poly-β(1-6)-*N*-acetylglucosamine (PNAG), partially deacetylated, positively charged, whose synthesis is mediated by the *icaADBC* locus. DNA sequences homologous to *ica* locus are present in many coagulase-negative staphylococcal species, among which *S. lugdunensis*, however, produces a biofilm prevalently consisting of proteins. The product of *icaA* is an *N*-acetylglucosaminyltransferase that synthetizes PIA oligomers from UDP-*N*-acetylglucosamine. The product of *icaD* gives optimal efficiency to IcaA. The product of *icaC* is involved in the externalization of the nascent polysaccharide. The product of *icaB* is an *N*-deacetylase responsible for the partial deacetylation of PIA. The expression of *ica* locus is affected by environmental conditions. In *S. aureus* and *S. epidermidis ica*-independent alternative mechanisms of biofilm production have been described. *S. epidermidis* and *S. aureus* undergo to a phase variation for the biofilm production that has been ascribed, in turn, to the transposition of an insertion sequence in the icaC gene or to the expansion/contraction of a tandem repeat naturally harbored within *icaC*. A role is played by the *quorum sensing* system, which negatively regulates biofilm formation, favoring the dispersal phase that disseminates bacteria to new infection sites. Interfering with the *QS* system is a much debated strategy to combat biofilm-related infections. In the search of vaccines against staphylococcal infections deacetylated PNAG retained on the surface of *S. aureus* favors opsonophagocytosis and is a potential candidate for immune-protection.

## Introduction

The recognition of “slime” as a mucilaginous material elaborated by certain microrganisms such as molds and bacteria represents a very early discovery in the history of microbiology. Impressively, the first papers describing the ability of bacterial species to form slime date back to the beginning of the 20th century. The description of specific extracellular polymeric components that structurally contribute to slime composition started with the second half of the century, when Wilkinson (1958) and Catlin and Cunningham (1958) began to report the existence of extracellular polysaccharides and deoxyribonucleic acids.

The medical importance of bacterial biofilm was for the first time enlighten by Bill Costerton, recognized as the “Father of Biofilm,” who, in 1978 established an extraordinarily new microbiological paradigm, the “biofilm theory.” In an article, published in Scientific American, he asserted that bacteria stick on available surfaces in glycocalyx-enclosed biofilms and that the sessile bacterial population becomes predominant particularly in medical ecosystems (Costerton et al., 1978). Costerton's observations shifted the medical research from the attention to microbial cell-wall structures, which are the interface of planktonic bacteria with the environment, to the biofilm, which is the interface of sessile bacteria with their environment (Costerton, 1989). The introduction of the *biofilm theory* opened two lines of research: the study of biochemistry and genetics of biofilms and their formation and, on the other side, the improvement of the medical diagnosis and treatment of biofilm-centered infections.

The profound influence that the Costerton's insight exerted on the bio-molecular knowledge of bacterial adhesion and on the need of appropriate medical methods for diagnosis and treatment of biofilm-related infections is analyzed in a review written by one of us together with the Director of the Costerton's Institute. The review was thought in the sad circumstance of the passing away of Bill, remembered as a charming *Maestro* for a large number of colleagues and students (Ehrlich and Arciola, 2012).

The early experimental works on slime producing bacteria were just a prelude to the more elaborated concept of biofilm (Mack et al., 1975; Costerton et al., 1978), where the sessile life-cycle phase was progressively associated to the social behavior of communicating and mutually interacting bacterial cells (Stoodley et al., 2002), forming communities encased within a protective extracellular matrix (Costerton et al., 1987) derived from the elaboration of an exocellular slime (the glycocalix) (Gristina and Costerton, 1984).

Over the years, progressive light was cast on the importance of glycocalix elaboration and biofilm formation not only as a fundamental mechanism of adhesion and colonization of biomaterial surfaces (Jacques et al., 1987), but also as a critical virulence mechanism enabling bacteria to escape the host immune-response and resist medical antibiotic chemotherapies (Gilbert et al., 1997; Costerton et al., 1999). The possibility for bacteria with a planktonic phenotype, otherwise susceptible to the host defenses and medical treatments, to switch to a sessile form of life more adapt to survive to the aggressive environment of host tissues, assumed a specific meaning in the pathogenesis of clinical infections, especially those associated to implant materials.

The great difficulty to eradicate microbial infections generated by biofilm-forming bacteria in presence of implant materials has received increasing attention over the last decades. Indeed, once established on a biomaterial surface, biofilm-forming strains are capable to resist and survive common antibiotic regimens formulated against and active on planktonic bacteria.

Soon after the launch of the *biofilm theory*, *Staphylococcus epidermidis* was early discovered to be one of the principal actors of biomaterial-associated infections and, certainly, its ability to colonize implant surfaces and produce resistant biofilms was recognized as a key factor in determining its success.

With the studies on slime/biofilm production by clinical strains, the biofilm was progressively demonstrated to be an important mechanism in bacterial adherence and pathogenesis of infections associated to biomaterials surfaces (Christensen et al., 1982). The production of biofilm was searched both by phenotypic methods, such as the microtiter plate (MtP) test (Christensen et al., 1985), the Congo red agar (CRA) plate test (Freeman et al., 1989) and its optimization (Arciola et al., 2002b), and by molecular detection of the *ica* locus, that had been identified as the genetic basis of biofilm production in *S. epidermidis* (Heilmann et al., 1996). The presence of *ica* locus in *S. epidermidis*, detected together with the positivity of phenotypic evidence of biofilm production, was then proposed as a marker of virulence of *S. epidermidis* strains responsible for implant-associated infections (Arciola et al., 2002a,b). PIA, besides described in *S. epidermidis* strains (Mack et al., 1996), was also described in *S. aureus* and the role of *ica* locus recognized also in this species (Cramton et al., 1999). The *ica* locus was then found widespread present in biofilm producing *S. aureus* strains responsible for catheter and implant infections (Ammendolia et al., 1999; Montanaro et al., 1999; Arciola et al., 2001).

The great attention that staphylococcal biofilms have received in recent years is justified by the large prevalence of *S. aureus* and *S. epidermidis*, but also of other emerging coagulase-negative staphylococci, as etiological agents of implant-infections. For instance, in orthopedics epidemiological studies have shown that staphylococci are the primary cause of implant-infections, causing nearly 80% of all prosthetic infections (Arciola et al., 2005, 2014; Montanaro et al., 2011).

Bacteria in biofilms can resist antibiotics at concentrations up to 1000 times higher than those active on the same bacteria in the planktonic state (Ceri et al., 1999). Antibiotic substances targeting the biofilm phenotype are therefore urgently needed. In search for the most efficacious antibiotic treatments active on biofilms, it has become common to assay antibiotic substances not only on planktonic bacteria to extrapolate minimal inhibitory concentrations (M.I.C.), but also screen them on sessile bacteria to achieve relevant information on the minimum biofilm eradication concentration (M.B.E.C.) (Ceri et al., 1999; Parra-Ruiz et al., 2010).

Apart from the efforts to identify the most active combinations of conventional antibiotics to eradicate bacterial biofilms, increased attention is being paid to identify new antibacterial molecules specifically targeting the biofilm state and therefore defined anti-biofilm substances. The great interest to counteract biomaterial-associated infections caused by biofilm-producing bacterial species has therefore seen the proliferation of studies screening different classes of compounds ranging from herb extracts, natural antimicrobial peptides, molecules of microbial origin to synthetic molecules. The intent has become not just the treatment but also the prevention of biofilm formation. This important goal can be achieved by the better knowledge of the molecular mechanisms of biofilm production and control and by the development of anti-biofilm biomaterial surfaces (Arciola et al., 2012; Campoccia et al., 2013). In this context, the knowledge of biofilm pathophysiology plays a fundamental role, as different strategies can be developed to contrast bacterial colonization and biofilm formation. They all rely on a deep understanding of the mechanisms implicated in biofilm production and of the fine mechanisms that rule the expression of the biofilm phenotype and govern biofilm formation. Two main anti-biofilm strategies are presently under consideration. One is based on biofilm disaggregating agents, such as enzymes that attack the PNAG (Dispersin B) or the extracellular DNA (DNase I) or the biofilm proteins (proteinase k) (Arciola, 2009; Kaplan, 2009; Arciola et al., 2012).

The other strategy is based on the modulation of the effector molecules, an approach called *quorum quenching* (QQ), which consists in interfering with the intercellular bacterial communications, with the aim at artificially inducing bacteria to assume a planktonic rather than sessile phenotype. In other words, the latter target consists in fooling the *quorum sensing* (QS) system. Based on signal molecules often referred to as *pheromones* or *autoinducers*, the QS system enables bacteria to sense their own density in the *milieu* and modify their phenotype accordingly. This involves ruling the expression of distinct traits for the specific cellular phase of growth and/or *milieu* colonization.

While some of the principal components of the QS system in *S. aureus* and *S. epidermidis* have been unveiled, much still remains to be elucidated as significant functional differences exist between the two bacterial species and, even, among different strain types, which often happen to compete in the environment just using different allelic forms of the signal molecules. These different alleles of signal molecules often exert the function of QS interference in the competition among different types of strains.

A brief description of some fundamental aspects of staphylococcal biofilm pathophysiology concerning the ultrastructural composition of the extracellular biofilm matrix, and the genetic mechanisms governing biofilm formation will be depicted in the next paragraphs.

## Chemistry of PIA and alternative forms of biofilm

The investigation of the chemical nature of the extracellular biofilm matrix began rather early around the years ‘50 s. However, in spite of many efforts made to elucidate its composition, the difficult purification of bacterial extracellular matrix and the multiplicity of analytical techniques adopted, often generating artifacts, initially led to divergent results. Thus, depending on their different experimental approach, different authors described different main exopolysaccharide components: the capsular polysaccharide-adhesin (PS/A) (Muller et al., 1993), the polysaccharide intercellular adhesin (PIA) (Mack et al., 1996), poly-β(1-6)-*N*-acetylglucosamine (PNAG) (Maira-Litrán et al., 2002), and *S. aureus* exopolysaccharide (SAE) (Joyce et al., 2003). In 2005, was finally demonstrated that PIA from *S. epidermidis* was structurally identical to the poly-β(1-6)-*N*-acetylglucosamine from the PS/A-overproducing strain *S. aureus* MN8m (Sadovskaya et al., 2005), definitively solving a dilemma lasted for more than a decade on the real chemical formula of the main exopolysaccharide component of the biofilms of most *S. aureus* and *S. epidermidis* clinical strains.

Currently, poly-β(1-6)-*N*-acetylglucosamine, alternatively named with the synonymous terms PIA or PNAG, has therefore been identified as the main exopolysaccharide component of staphylococcal biofilm matrix. Interestingly, the same exocellular polysaccharide has recently been identified even in numerous other gram-negative bacterial species members of the *Proteobacteria* family, including *Escherichia coli*, *Yersinia pestis*, *Pseudomonas fluorescens*, *Bordetella* spp., *Xenorhabdus nematophila*, *Aggregatibacter actinomycetemcomitans*, and *Actinobacillus pleuropneumoniae* (Ganeshnarayan et al., 2009), suggesting a convergent evolution in phylogenetically diverse bacteria.

Although PIA certainly represents a main mechanism of biofilm formation in *S. aureus* and *S. epidermidis*, numerous advancements in the study of biofilm have shown the existence, especially for *S. aureus*, of alternative forms of biofilm that are PIA-independent. The observation that a minor proportion of *S. aureus* strains can form biofilm even in the absence of the *ica* locus and that certain strains carrying such locus continue anyway to produce biofilm even after deletion of the locus suggested the existence of *ica*-independent pathways (O'Gara, 2007). The assumption of the possibility of alternative mechanisms to produce biofilms was long debated despites some very early studies had already suggested the complexity of biofilm architecture and the inclusion of extracellular polymeric substances different than PIA, such as e-DNA and teichoic acids, in the biofilm matrix.

A number of proteins localized in the extracellular matrix of biofilms have been identified that can generate PIA-independent biofilms. The biofilm-associated protein (Bap), a 2276-amino acid surface protein, is one of these proteins enabling biofilm production even in the absence of production of the exopolysaccharide component (Cucarella et al., 2004; Tormo et al., 2005). The Bap protein seems to play a role prevalently in human staphylococcal infections caused by coagulase-negative staphylococcal (CoNS) species. The *bap* gene encoding for Bap has been identified in *S. epidermidis*, *S. chromogenes*, *S. xylosus* and a few other CoNS species, where it is often carried in a putative composite transposon (Cucarella et al., 2004; Tormo et al., 2005). However, as far as *S. aureus* is concerned, the role of Bap seems less relevant in isolates from human infections (Vautor et al., 2008; Tang et al., 2013) and, up to now, exclusively strains of veterinary origin (e.g., bovine mastitis) have been found to harbor the *bap* gene (Valle et al., 2012). However, the functions of Bap are still far from being totally understood and are not limited to biofilm formation. Indeed, it has been found that Bap not only promotes the adhesion, but also prevents the entry of *S. aureus* into epithelial cells (Valle et al., 2012). Bap involvement in the pathogenesis of infections is therefore more complex as this protein influences the extent of bacterial internalization into host cells and consequently tissue invasiveness.

In the production of biofilm more clinically significant is the role of another protein factor, namely the accumulation associated protein (Aap). Among biofilm-forming *S. epidermidis* isolates from total hip or total knee infected arthroplasties, up to 27% of the strains were found to be endowed with this surface protein and negative to the *ica* locus (Rohde et al., 2007).

Differently, surface proteins such as SasG (Geoghegan et al., 2010), SasC (Schroeder et al., 2009), Protein A (Merino et al., 2009), and two fibronectin-binding proteins, namely FnBPA and FnBPB (O'Neill et al., 2008), have been documented to contribute to biofilm formation in *S. aureus*. Interestingly, *S. aureus* clinical strains are all generally endowed with the *ica* locus. These alternative mechanisms of biofilm formation can probably concur and could be switched on in different phases of the pathogenesis of infections, adapting the characteristics of the biofilm extracellular matrix in response to external stimuli (Houston et al., 2011), in order to colonize and establish the infection in host tissues, while evading the immune response and the effects of antibiotic treatments.

The protein components of staphylococcal biofilms are discussed in depth in a recent review that illustrates how a multitude of proteins intervene at different stages of the biofilm formation, with certain proteins contributing to the biofilm accumulation and others mediating the primary attachment to the surfaces (Speziale et al., 2014).

## The synthesis of PIA

PIA, as described above, is now well-established to consist of poly-β(1-6)-*N*-acetylglucosamine, a linear glucosaminylglycan that plays a fundamental function in mediating intercellular adhesion of bacterial cells. In addition to bacterial aggregation, this exopolysaccharide has important structural functions in the biofilm matrix architecture, and is implicated in bacterial adhesion to biomaterial surfaces as well as evasion from host immune-response (Vuong et al., 2004). PIA synthesis is mediated by the *icaADBC* locus, first discovered and investigated by Heilmann et al. (1996). The *icaADBC* locus was initially described in *S. epidermidis*, but few years later its presence was confirmed even in many other species of the *Staphylococcus* genus, *S. aureus* included (Cramton et al., 1999). The *ica* locus is however part of the “accessory genes” genome and not of the so-called “core” genome, meaning that it is not found in all bacterial strains. Its presence is exclusively observed as part of the virulon of exopolysaccharide-based biofilm-forming staphylococcal strains. In early studies, the *ica* locus was reported in a large proportion of staphylococcal strains isolated from implant related-infections (catheter-associated infections) (Arciola et al., 2001). Important differences in the prevalence of *ica*-positive strains had previously been observed also comparing clinical isolates (blood cultures) with saprophytic strains (85% vs. 6%) (Ziebuhr et al., 1997).

Further evidence of the important role of PIA as a virulence factor came also from experimental studies in rat models of intravascular catheter-associated infections with isogenic mutant strains deficient in PIA production (Rupp et al., 1999, 2001).

The *icaADBC* locus consists of four genes. The first two genes of this gene cluster, respectively *icaA* and *icaD*, exert a primary role in the exopolysaccharide synthesis. The former gene encodes for a transmembrane enzyme with *N*-acetylglucosaminyl transferase activity, necessary for the synthesis of the poly-*N*-acetylglucosamine polymer. However, the enzymic activity of the product of *icaA* becomes significant and olygomers longer than 20 residues are synthesized only when coexpressed with the product of the *icaD* gene (Gerke et al., 1998). Conversely, the product of the *icaC* gene appears to translocate the poly-*N*-acetylglucosamine polymer to the bacterial cell surface, while the *icaB* product operates the deacetylation of the molecule (Vuong et al., 2004). Deacetylation of poly-*N*-acetylglucosamine polymer is relevant for the structural development of exopolysaccharide-based biofilm, enabling the fixation of the polymer to the outer bacterial surface. The negative regulator termed intercellular adhesin locus regulator (*icaR*) gene governs the expression of the *ica* locus under the influence of SarA and the stress sigma σ^B^ (Cerca et al., 2008).

**Figure 1:**
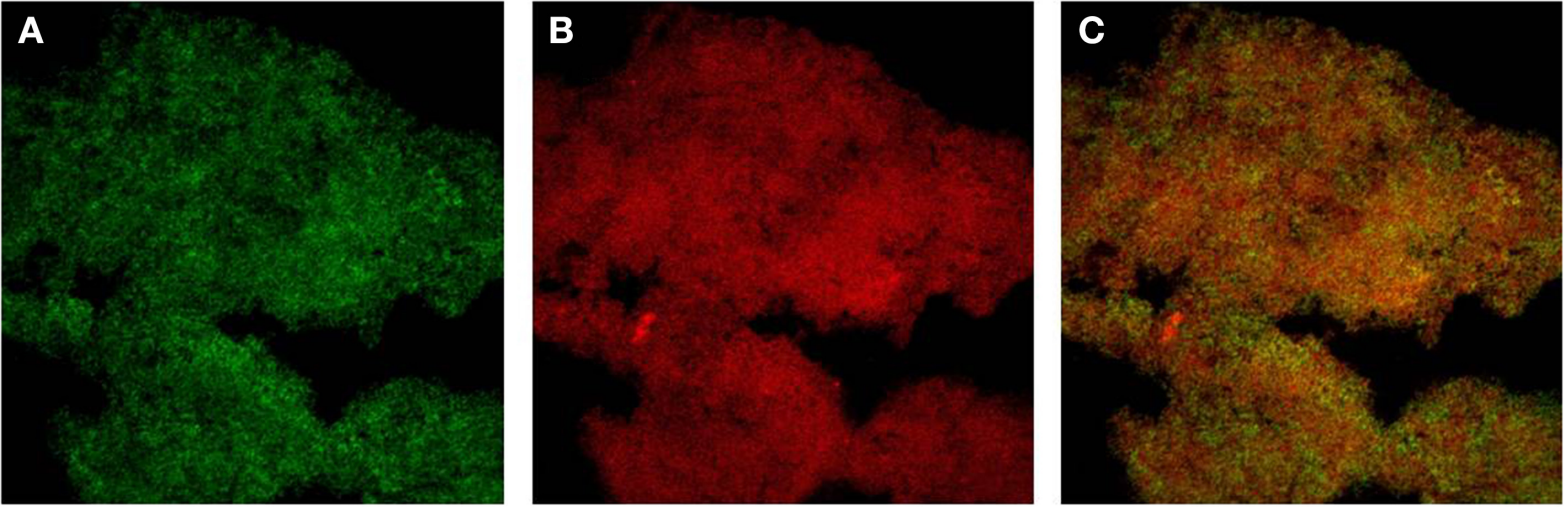
**Double staining with FITC-Wheat Germ Agglutinin (FITC-WGA, for exopolysaccharide detection) and SYPRO Ruby (FilmTracer™ SYPRO® Ruby Biofilm Matrix Stain, for protein detection) was carried out as described in Ravaioli et al. (2012)**. **(A)** Green channel image showing PNAG stained with FITC-WGA. **(B)** Red channel image showing the proteic component stained with SYPRO® Ruby. **(C)** Merged image of the two channels.

While DNA-sequences homologous to the *ica* locus have been identified also in many staphylococcal species other than *S. aureus* and *S. epidermidis*, up to now they have not been reported for two staphylococcal species, respectively *S. haemolyticus* and *S. saprophyticus*. Species such as *S. lugdunensis*, although endowed with the *ica* locus, appear to produce a biofilm whose matrix prevalently consists of protein material (Frank and Patel, 2007). By CLSM and specific staining with fluorescent dyes we have observed that the biofilm of *S. lugdunensis* is composed of both PIA, which is stained with FITC-WGA, and proteins, stained with Sypro®-Ruby, specific for the protein component (Figure 1).

## Phase variation in staphylococci for the biofilm formation

An interesting question is the phase variation of staphylococci, which consists in a process of switching on/off for the expression/silencing of the *ica* locus, leading to a quantitative variation of biofilm production. In *S. epidermidis*, the phase variation for biofilm formation is thought to be a mechanism of persistence and relapse. The insertion/excision of the insertion sequence IS*256* in the gene *icaA* or in *icaB*, or, more frequently, in *icaC* as a possible mechanism of the phase variation was hypothesized by Ziebuhr et al. (1999). These Authors observed the phase variation in two *S. epidermidis* reference strains that had been subjected to repeated *in vitro* subcultures. In a different way, the natural occurrence of the IS*256* insertion element either in *ica* locus or in the genomic DNA of clinical strains of *S. epidermidis* - just as they had been isolated, that is without artificial manipulations to induce phase variations - was searched by Arciola et al. (2004). The search for *ica* genes was carried out in 120 *S. epidermidis* isolates from prosthesis-associated infections and in 4 *S. epidermidis* reference strains and was compared with the bacterial phenotypes (ability/inability to produce biofilm). Moreover, two biofilm-negative RP62A-derived acriflavin mutants (D9 and HAM892) were analyzed. The four genes of the *ica* locus appeared, in all cases of that collection, strictly linked each other, so that they were either all present or all absent, nor were detected gene deletions within the *ica* locus. IS*256* was present in eight out of the 69 *ica*-negative strains and in 34 out of the 51 *ica*-positive strains. However, when IS*256* was found in the bacterial genomic DNA, it was never found within the *ica* locus, this observation suggesting that the insertion/excision of IS256 is not a natural occurring mechanism for off/on switching the biofilm production. In the same study, for the first time, two RP62A-derived acriflavin mutants, D9 and HAM892, unable to produce biofilm, were shown to harbor within their *icaC* genes, at the base 3319, a 1300-bp insertion corresponding to IS*256*. Although the insertion was found within *icaC*, as in the study of Ziebuhr et al. (1999), it was traced in a position rather different from that described by Ziebuhr et al. (1999). The different point of insertion was ascribed by the Authors to the different mutagenesis conditions, namely repeated subcultures in the experiments reported by Ziebuhr et al. (1999) and, instead, the chemical mutagenesis by acriflavin in the study of Arciola et al. (2004). However, interestingly, in none of the 120 *S. epidermidis* clinical isolates from prosthesis associated infections the IS256 insertion element was found within the *ica* operon, neither in some strains that, although *ica*-positive, were biofilm *non*-producers. Thus, the insertion/excision of IS*256* in *ica* operon does not appear as a natural occurring mechanism for switching off/on the biofilm production, but rather as the consequence of either a chemical mutagenesis or of manipulative mutations. Afterwards, in the same 2004 year, only some months later, the phase variation of biofilm formation by an insertion sequence was described also in *Staphylococcus aureus* by Kiem et al. (2004). A biofilm-negative phase-variant *S. aureus* mutant was detected from six strains subjected to repeated subcultures. Again, the *ica*C gene of the phase-variant strain was found to be inactivated by the insertion sequence IS*256*.

More recently, a mechanism of phase variation of the poly-N-acetylglucosamine expression in *Staphylococcus aureus* has been described that does not involve the insertion/excision of IS*256* but the expansion or contraction of a simple tetranucleotide tandem repeat housed within *icaC*. Inactivation of IcaC by the expansion or contraction of this tetranucleotide tandem repeat results in a PIA/PNAG-negative phenotype (Brooks and Jefferson, 2014). And indeed, the expansion (or contraction) of a 4-nt tandem “ttta” repeat shifts the reading frame of *icaC* and leads to a premature stop codon, truncating the IcaC protein at 303 amino acids, 47 amino acids shorter than the full-length protein. The Authors suggest that, under certain conditions, the loss of PIA/PNAG production may be advantageous during infection. Inactivation of *icaC* is therefore a mechanism of phase variation for PIA/PNAG expression and *icaADB* may contribute to the bacterial fitness, by a mechanism still unknown and involving the absence of an intact *icaC* gene and of PIA/PNAG production. All these findings indicate that the mutation of i*caC* is the preferred “off switch” for PIA/PNAG production.

## Genetic control of biofilm metabolism

The numerous studies on the genetic control of biofilm production in staphylococci have led to consider the expression of the biofilm forming phenotype very complex. This complexity is in part because there is a multiplicity of factors contributing to the biofilm extracellular matrix, these varying with the bacterial species but also, within the same species, with the strain type. In addition, biofilm production derives from a complicated equilibrium of production of extracellular polymeric substances, including amyloid fibrils and polymerized phenol soluble modulins (PSMs), and their catabolism determined by expression of enzymes such proteases, nucleases and soluble, still non-polymerized, PSM peptides (Schwartz et al., 2012).

A fine control of sessile and planktonic phenotypes is highly required to explicate a well coordinate and efficacious action during the invasive phase. The expression of biofilm is therefore governed by mechanisms of collective coordination. These mechanisms enable not only the sensing of environmental stimuli, but also the density of bacterial cells belonging to the same group and sharing the same pheromone system.

Diverse studies have documented as *ica*-positive *S. epidermidis* easily express their biofilm-forming phenotype under *in vitro* conditions. Differently, in *S. aureus*, whose strains are generally all endowed with *ica* locus, the biofilm production is not always fully expressed *in vitro* and often requires a modified atmosphere (anaerobiosis) or supplementation of the growth broth with nutrients in order to be fully manifested (Arciola et al., 2001; Cramton et al., 2001; Stepanović et al., 2003, 2007).

*Vice versa*, *S. aureus* strains manifestly express increased biofilm production *in vivo* (Ammendolia et al., 1999; O'Gara, 2007). *In vitro* stress conditions induced by iron limitation, starvation, thermal stress or subinhibitory concentrations of ethanol, salt and some antibiotics have also been found to increase the amount of biofilm produced.

Therefore, a series of stimuli from the environment and from bacterial density are sensed by bacterial cells and influence the expression of biofilm-forming phenotype. As far as the response to environmental stimuli is concerned, important regulators of staphylococcal biofilm production are represented by the two-component signal transduction systems (TCSs) and σ^*B*^. TCSs mediate a diverse range of adaptive responses in response to environmental stresses. First identified in *S. aureus* (Fournier and Hooper, 2000), the presence of ArlRS TCS was later confirmed also in *S. epidermidis* (Zhu et al., 2010). Recent data show that ArlRS plays an important role in the regulation of *S. epidermidis* biofilm formation, and acts in an *ica*-dependent manner distinct from the role of ArlRS in *S. aureus* biofilm formation, which was found to be *ica*-independent (Wu et al., 2012). In addition to influencing biofilm formation, ArlRS is also involved in the modulation of bacterial autolysis and, consequently, of e-DNA release contributing to the biofilm extracellular matrix.

In *S. aureus* another two-component regulatory system is the *lytSR* operon that affects murein hydrolase activity and autolysis (Brunskill and Bayles, 1996). The LytS sensor component, interacting with its cognate response regulator LytR, activates the transcription of genes under its control. The target of the system is the *lrg/cid* operon, which has been shown to be a regulator in the control of cell death and lysis during biofilm development (Rice et al., 2003; Rice and Bayles, 2008). The *cidA* gene encodes a putative holin protein that is an effector of murein hydrolase activity and cell lysis, while *lrgA* encodes a putative antiholin that is an inhibitor of these processes (Rice et al., 2007), Thus, the biological function of the *cid* and *lrg* operons is to provide a source of extracellular genomic DNA (eDNA) for interweaving and strengthening with this molecule the scaffold of the matrix of biofilm.

**Figure 2:**
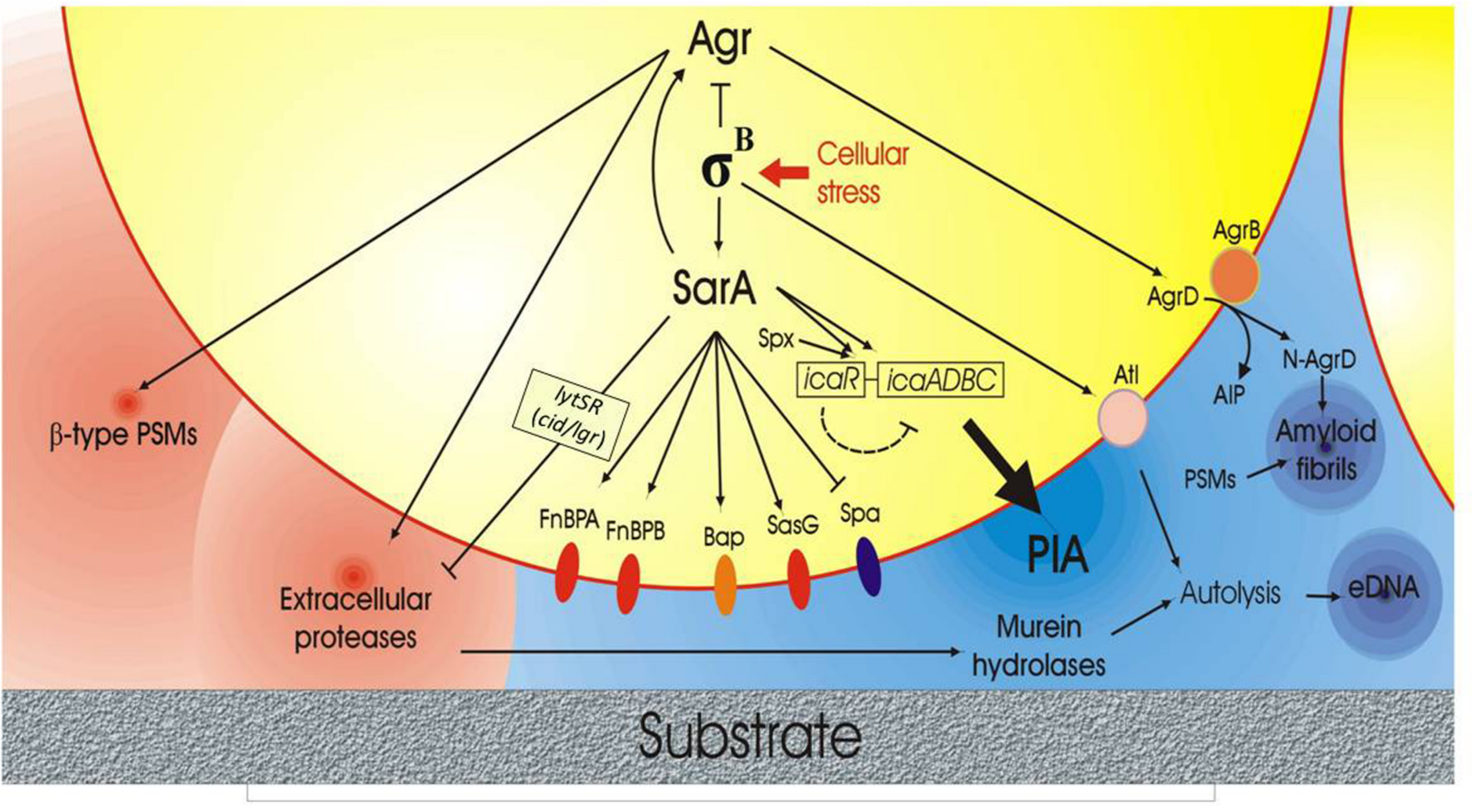
**Scheme of the complex network of interactions governing biofilm formation and disruption in *S. aureus* based on current scientific evidences**. The right side of the figure illustrates the anabolic phase of biofilm with the production of some fundamental extracellular polymeric substances (EPS) such as PIA, extracellular-DNA (eDNA) and amyloid fibrils. The *lytSR* operon with its target genes *lrg/cid*, which affects murein hydrolase activity, is also represented. The center of the figure reports the protein membrane components implicated in biofilm formation, these including the FnBPs adhesins, the Biofilm associated protein (Bap), SasG and Spa. Conversely, in the left side of the figure, the molecules playing a role in biofilm catabolism and extracellular biofilm matrix disruption, such as phenol-soluble modulins (PSMs) and extracellular proteins, are reported. Agr QS system, σ^B^ factor and SarA appear to act as central regulators, orchestrating the bacterial behavior in response to stress factors, cellular densities and cell cycle phases.

The complex intervening factor influencing biofilm production and, in particular PIA synthesis, are depicted in Figure 2.

It has been earlier anticipated that biofilm is also influenced by bacterial density. Intercellular communication in staphylococci is enabled by the so-called *quorum sensing* (QS) systems.

The role of QS system in controlling staphylococcal biofilm dispersal and structuring is presented in details in another review of this series (Le et al., 2014) and will be treated here only summarily.

In staphylococci, the system that regulates the expression of virulence factors in response to cell density is the accessory gene regulator (*agr*) system (Recsei et al., 1986; Novick et al., 1993). The importance of the *agr* in biofilm formation in both *S. aureus* and *S. epidermidis* was first shown by the group of Michael Otto (Otto, 2001), who highlighted that, while *agr* leads to increase expression of toxins, serine protease, DNase, fibrinolysin, enterotoxin B, and toxic shock syndrome toxin-1, it decreases the expression of colonization factors and biofilm formation. Since biofilm is one of the most important virulence factors in chronic bacterial infections, the use of *agr*-inhibiting substances for anti-staphylococcal treatment has been proposed, but it may have severe drawbacks, as it might turn an acute into a chronic infection by promoting the expression of colonization factors (Otto, 2001).

Among the peptides strictly controlled by the *agr* locus, a family of short staphylococcal peptides, Phenol-Soluble Modulins (PSMs), characterized by amphipathic a-helical structure conferring surfactant-like properties, have been shown to be key effector molecules in biofilm structuring, dispersal and dissemination, by a mechanism consisting in the disruption of non-covalent interactions between biofilm matrix macromolecules (Otto, 2013). These peptides have been demonstrated to be relevant in the pathogenesis of *S. aureus* and *S. epidermidis* biofilm-associated infection, under both *in vitro* and *in vivo* models (Le et al., 2014).

## The debated promise of quorum quenching (QQ)

In the introduction, quorum quenching (QQ), alternatively known as quorum interference, has been mentioned as one of the latest strategies to counteract staphylococcal infections. In nature, QQ is a strategy pursued by antagonistic bacterial strains competing for the environment (Geisinger et al., 2009). QQ can be achieved by alternative tactics, these including quorum sensing disruption through inhibition of signal molecule biosynthesis, signal molecule inactivation and blockade of signal transduction (Kiran et al., 2008; Rampioni et al., 2014). A weakness of the QQ approach resides in the fact that not only QS systems markedly differ between Gram-positive and Gram-negative bacteria, but also their functions are different in the same *genus* and sometimes even in diverse strain types of the same species (Geisinger et al., 2012). This means that it may result hard to find universal key molecules able to exert their desired action on all pathogens and that what can inhibit biofilm-formation in a species could stimulate it in another.

However, while inhibiting *agr* would be counterproductive and the use of QS inhibitors is debatable for combatting biofilm-associated infections by staphylococci, *vice versa*, QS interference is expected to become a powerful strategy to control virulence and antibiotic tolerance of Gram-negative bacteria.

To date, methods that can be used to disrupt quorum sensing include: (1) antagonizing signal binding to LuxR-family receptor, (2) inhibiting signal production, (3) degrading signals, (4) trapping signals, and (5) suppressing synthase and receptor activities, stabilities or productions (Hirakawa and Tomita, 2013).

Overall QQ appears an important strategy for applications in medicine (but also veterinary and agriculture), opening new horizons for preventive/therapeutic measures alternative or adjuvant to conventional antibiotics (Costerton et al., 2007).

## PIA in immunotherapeutic strategies to combat staphylococcal infections

The poly-*N*-acetyl-β-(1,6)-glucosamine, PNAG, also designed as PIA, is a high-profile candidate for a vaccine that could possibly provide protection against both coagulase-positive and coagulase-negative staphylococci. As outlined above, the native form of PIA/PNAG is partially de-*N*-acetylated (dPNAG). It has been observed that only antibodies to the deacetylated epitopes of PNAG are able to give protection, favoring opsonization of *S. aureus* and killing by human neutrophils (Kelly-Quintos et al., 2005, 2006).

Antibodies that bind to PNAG with either low (<15%) or high (>90%) levels of acetate were shown to have a superior opsonic and protective activity than antibodies that bind to PNAG with only high levels (>70%) of acetate (Cerca et al., 2007). Both in *S. epidermidis* and in *S. aureus*, IcaB is a deacetylase that causes partial deacetylation of PNAG and thus ensures a better surface retention of PNAG and optimal biofilm formation.

By studying the acetylation of PNAG by *icaB* negative *S. aureus* mutants, Cerca et al. (2007) observed that less PNAG was associated to the bacterial surface, and this strain was highly susceptible to antibody-independent killing by neutrophils. A *S. aureus* mutant with over-expression of *icaB* producing primarily surface-associated PNAG, was more susceptible to opsonophagocytosis with antibody to deacetylated PNAG. The higher retention of deacetylated PNAG on the surface of *S. aureus* provides a molecular mechanism explaining the superior opsonic and protective activity of antibody to dPNAG (Cerca et al., 2007).

While there is a large consensus on the need of a vaccine protecting people from staphylococcal infections, there is much less clarity about the choice of efficacious candidates for a component vaccine. Projan et al., in discussing the possible targets of an anti-*Staphylococcus* vaccine, list almost eight diseases and about thirty possible molecular targets (Projan et al., 2006).

The choice of an appropriate antigen for immunotherapy should attentively consider the issue of the immune evasion. In fact, PIA/PNAG, in addition to its role in intercellular adhesion and biofilm formation, has been pointed out to play a role just in the immune evasion by bacteria. Evidence suggests that antibodies against PIA/PNAG often recognize the secreted PIA/PNAG rather than its surface-associated form, this behavior resulting in an ineffective immune response (Cerca et al., 2007). An effective immune response against surface-associated PIA/PNAG has been evoked by a conjugate vaccine, composed of the *Staphylococcus aureus* PNAG and Clumping Factor A, which has been proved successful in eradicating infection (Maira-Litrán et al., 2012). Thus, PIA/PNAG protects the bacteria from immune defenses but, otherwise, it could actually be the target of an effective immune response.

Although this field is rich of interests and researches, and progress have been made, further experimental, epidemiological, pre-clinical and clinical studies are required before an efficacious vaccine could be achieved.

### Conflict of interest statement

The authors declare that the research was conducted in the absence of any commercial or financial relationships that could be construed as a potential conflict of interest.
